# Dataset of the AAC2 conformations in the c-, intermediate- and m-states obtained from free-energy simulations

**DOI:** 10.1016/j.dib.2016.04.022

**Published:** 2016-04-13

**Authors:** Adriana Pietropaolo, Ciro Leonardo Pierri, Ferdinando Palmieri, Martin Klingenberg

**Affiliations:** a Dipartimento di Scienze della Salute, Università di Catanzaro, Viale Europa, 88100 Catanzaro, Italy; b Department of Biosciences, Biotechnologies and Biopharmaceutics, University of Bari, Via E. Orabona 4, 70125 Bari, Italy; c Institut für Physiologische Chemie, Schillerstr.44, 80336 München, Germany

**Keywords:** AAC, AAC conformational states, Mitochondria, Mitochondrial carrier, Molecular dynamics, Transport mechanism

## Abstract

The data reported herein are related to the article entitled: “The switching mechanism of the mitochondrial ADP/ATP carrier explored by free-energy landscapes” (Pietropaolo et al., 2016) [1].

We report the coordinates of the ADP/ATP carrier (AAC2) in the presence and absence of adenine and guanine nucleotides in the c-, intermediate- and m-states obtained from the free-energy simulations and corresponding to the free-energy minima.

## Specification Table

**Table:** 

Subject area	*Chemistry, Biology*
More specific subject area	*Mitochondrial carriers*
Type of data	*Atomic coordinates, tab-limited text file*
How data was acquired	*Free-energy simulations*
Data format	*Analyzed PDB files*
Experimental factors	*NVT ensemble, T=300 K*
Experimental features	*These data regard the AAC2 coordinates obtained from well-tempered metadynamics free-energy simulations*
Data source location	*Catanzaro (IT), Bari (IT)*
Data accessibility	*The data are within this article*

**Value of the data**

• This is the first report presenting the structures of AAC2 in the c-, intermediate- and m-states in the presence and absence of adenine and guanine nucleotides.
• The data are useful to the readers interested in the conformational changes of transport proteins in general and of the ADP/ATP carrier, in particular, during substrate translocation.
• The data are valuable to gain insight into the transport mechanism of the ADP/ATP carrier and the other members of the mitochondrial carrier family at the molecular level.
• The data can be exploited in further studies, for example of molecular dynamics and site-directed mutagenesis, of the ADP/ATP carrier in the c-, intermediate- and m-states.

## 1 Data

Seven sets of AAC2 conformations in the presence and absence of adenine and guanine nucleotides have been obtained in the c-, intermediate- and m-states. These structures correspond to the free-energy minima disclosed through well-tempered metadynamics free-energy simulations and are herein reported as PDB files.

## 2 Experimental design, materials and methods

The initial structure of human AAC2 was obtained through a comparative modeling [2], [3] by using the bovine AAC1 structure (PDB code 1OKC) [4] as a template, owing to the high sequence homology with the crystallized bovine AAC1 (the human AAC2 shares more than 90% of identical residues with the bovine AAC1). The C-terminal portion ^295^KKYT^298^ of AAC2 not represented in the crystallized bovine AAC1 was subsequently added at the C-terminus after an initial equilibration of the segment by using the available crystallized structures of yeast AAC2 (pdb code 4C9H) and AAC3 (pdb code 4C9Q) as template-driving structures [5]. The derived model was inserted in a thermalized palmitoyl-oleoyl-phosphatidylcholine (POPC) bilayer consisting of 110 lipid units solvated in 9156 water molecules. The net excess charge of AAC2 in the absence of any nucleotide was neutralized adding 17 chloride counterions. The ATP, ADP, AMP, GTP, GDP or GMP nucleotides were inserted in the c-state, in the intermediate-state and in the m-state obtained through the preliminary empty AAC2 free-energy simulations, at the positions close to Y195 and Y191, close to K23, R80, R280, R236 and close to G183, Y187, I184, S228 and G225. The net excess charge of AAC2 was neutralized by 13 Cl^−^ counterions in presence of ATP and GTP, 14 Cl^−^ counterions in presence of ADP and GDP, and 15 Cl^−^ counterions in presence of AMP and GMP. Seven sets of well-tempered metadynamics free-energy simulations [6], [7] allowed the reconstruction of the free-energy landscapes [8], [9], [10], [11], [12] of the AAC2 switching from the c-state to the m-state [1].

The conformation states of AAC2 reported in this data article were selected among those present in each of the three free-energy basins associated to the c-, intermediate- and m-state [1]. First we clustered the conformations belonging to the three deepest free-energy minima and then chose the conformations on the basis of the ion-pair distances at the level of the c-gate and m-gate [4], [5], [13], [14], [15], [16]. Among all the conformations belonging to the c-basin, we selected the c-state as the one having the shortest ion pairs among the residues of the m-gate and the highest ion pairs among the residues of the c-gate. In a similar way, we selected the m-state, among the conformations belonging to the m-basin, as that having the shortest ion pairs among the residues of the c-gate and the highest ion pairs among the m-gate residues. For the intermediate-state, the conformation having the shortest ion pairs among the residues of both the c-gate and m-gate was selected among all the conformations belonging to the intermediate-basin.
