# Supplementary material for: Dataset of the AAC2 conformations in the c-, intermediate- and m-states obtained from free-energy simulations
Source: Data Brief. 2016 Apr 13;7:1355–7. doi: 10.1016/j.dib.2016.04.022 (PMC4845157; doi:10.1016/j.dib.2016.04.022)
Supplement: Supplementary file 1 — Supplementary material [file mmc1.pdf]

**Conflicts of interest: none**
